# High prevalence of HIV and syphilis and associated factors among low-fee female sex workers in mainland China: a cross-sectional study

**DOI:** 10.1186/1471-2334-14-225

**Published:** 2014-04-26

**Authors:** Chu Zhou, Keming Rou, Willa M Dong, Yu Wang, Wei Dong, Yuejiao Zhou, Xi Chen, Manhong Jia, Wei Liu, Jun Zheng, Yanling Ma, Youfang Li, Zunyou Wu

**Affiliations:** 1 National Center for AIDS/STD Control and Prevention, Chinese Center for Disease Control and Prevention, 155 Changbai Road, Beijing, Changping District 102206, China; 2 Guangxi Zhuang Autonomous Region Center for Disease Control and Prevention, Nanning, China; 3 Hunan Provincial Center for Disease Control and Prevention, Changsha, China; 4 Yunnan Provincial Center for Disease Control and Prevention, Kunming, China

**Keywords:** HIV, Syphilis, Sexually transmitted infections, Low-fee sex workers, China

## Abstract

**Background:**

The prevalence of HIV and syphilis among middle and high-fee female sex workers (FSWs) has been widely reported but little is known among low-fee FSWs. This study aims to determine the prevalence and associated factors of HIV and syphilis among low-fee FSWs in China.

**Methods:**

A cross-sectional study design was used. A convenience sample of low-fee FSWs was recruited from venues by outreach workers in 12 cities. Structured questionnaire interviews and blood sampling for HIV and syphilis were carried out. Univariate and multivariate logistic regression were used for assessing potential associated factors.

**Results:**

This study enrolled 781 low-fee FSWs. There were 37 (4.7%) HIV positive participants and 117 (15.0%) participants were infected with syphilis. Final multivariate analysis identified five factors associated with HIV infection: older age (OR:2.6, 95% CI:1.1-6.1), local household registration (OR:3.3, 95% CI:1.5-6.9), employed in Yunnan province (OR:2.7, 95% CI:1.1-6.7), soliciting in self-rented rooms and “market day” buildings (OR:3.9, 95% CI:1.5-10.0), injection drug use in the past 6 months (OR:13.5, 95% CI:4.5-40.1); and four factors associated with syphilis infection: older age (OR:1.8, 95% CI:1.2-2.9), employed in Yunnan province (OR:2.1, 95% CI:1.2-3.6), soliciting in self-rented rooms and “market day” buildings (OR:2.3, 95% CI:1.4-3.7) , and no consistent condom use with clients in the past 30 days (OR:1.6, 95% CI:1.0-2.6).

**Conclusions:**

A high prevalence of HIV and syphilis were found among low-fee FSWs. Those soliciting in self-rented rooms and “market day” buildings with the lowest income, and injection drug users (IDUs) in this population should take priority in further intervention strategies.

## Background

Female sex workers (FSWs) are one of the groups monitored through the national sentinel surveillance system due to a high risk of acquiring and transmitting HIV through heterosexual commercial sex in mainland China [1,2]. Some sexually transmitted infections (STI) such as syphilis are also included in sentinel surveillance as it is among the top five reported communicable diseases in major provinces and potentially facilitates HIV transmission [3,4]. Several tiers of FSWs have been delineated in the literature according to the price charged, work venues, and prestige [5,6]. Low-fee FSWs are described as those who work in smaller and hidden venues such as guesthouses, hair and beauty salons, on the street, and self-rented rooms [7]. In rural areas, low-fee FSWs may also solicit clients in small residential buildings near markets, and give a proportion of their earnings to the venue owner during “market day”, when local residents congregate for trading [8].

National sentinel surveillance data estimate that the average HIV prevalence among FSWs is less than 1% [9-12]. Data from cross-sectional studies show that low-fee FSWs have significantly higher HIV and syphilis prevalence compared with higher-fee FSWs [3,5,6,13-15]. A study conducted in 6 cities found that HIV prevalence was 1.37%, 0.28%, and 0.07%, among low, middle and high- fee FSWs, respectively [6]. Similarly, syphilis prevalence among low, middle and high-fee FSWs was 9.7%, 4.3%, and 2.2%, respectively [3]. Higher HIV and syphilis prevalence among this population are associated with disadvantaged socio-demographic status including older age, less education, and lower socioeconomic background, and low fee FSWs are less likely to perceive themselves to be at risk for HIV or use condoms with clients [16,17].

Due to the difficulty of recruiting low-fee FSWs, this group is typically underrepresented in FSW studies and surveillance compared to middle to high-fee FSWs [2,3,5]. As a result, risk behaviors and factors associated with HIV/STI infection for this group, as well as differences within this group, are not well-understood despite the evidence of increased HIV/STI risk among low-fee FSWs. Our study aims to conduct a multi-site survey among low-fee FSWs to understand the overall prevalence and factors associated with HIV and syphilis, and to inform targeted intervention strategies.

## Methods

### Study design and study participants

This cross-sectional study was carried out in 12 cities across three provinces: Liuzhou, Guigang, Pingnan, and Du’an in Guangxi (located in southern China); Dali, Jinghong, Kaiyuan, and Menghai in Yunnan (located in southwest China); and Zhangjiajie, Jishou, Lingling, and Jianghua in Hunan (located in central China). We selected these cities based on three criteria: a high number of HIV cases acquired through heterosexual transmission reported to the national surveillance system in 2011; past experience conducting HIV/STI interventions for FSWs by local outreach workers; and input from provincial Centers for Disease Prevention and Control (CDC) on areas with rapidly expanding HIV epidemics among FSWs.

From November 2012 to January 2013, a convenience sample of low-fee FSWs was recruited through the following procedures: first, trained outreach workers from the local CDC, township or community hospitals identified and mapped all places where low-fee FSWs gathered. The venues included guesthouses, hair/beauty salons, streets, self-rented rooms and “market day” buildings. Township or community hospital clinicians were included as outreach workers since they were more familiar with location of self-rented rooms or “market day” venues. Then, different strategies were utilized to approach the participants. For FSWs who reported to a manager, outreach workers contacted the venue managers of the guesthouses, hair/beauty salons and street for permission to conduct the survey, and then approached participants directly in the venues after receiving permission; independent low-fee FSWs who solicit in self-rented rooms or “market day” buildings were approached directly by the outreach workers. Study staff introduced the survey, and invited eligible low-fee FSWs to participate. The eligibility criteria of participants in this study were: age 16 or above, had exchanged vaginal sex for money or a gift in the past 30 days, charged less than 50 RMB (~8.1 USD) for vaginal sex, were currently living and trading sex locally, and were able to give informed consent.

### Data collection

All participants were anonymously interviewed using a structured questionnaire by trained outreach workers in a private place in the venues. Socio-demographic characteristics collected included age, education level, marital status, ethnicity and household registration; work-related information included change in work location, length of time working as an FSW, venues, price charged for vaginal sex, provinces of employment and estimated average age of clients; HIV related behaviors included condom use with clients, injection drug use in the past 6 months, and HIV testing history in the past year.

Blood collection and pre-test counseling for HIV and syphilis were provided. All blood samples were tested for HIV and syphilis infection. Screening for HIV was carried out with enzyme-linked immunosorbent assays (ELISA) (Kinghawk Pharmaceutical Co., Ltd., Beijing, China), and positive screening results were confirmed with western blot (WB) confirmatory tests (MP Biomedicals Asia Pacific Pte Ltd., Singapore). Syphilis infection was screened for with the Rapid Plasma Reagin (RPR) test (Kinghawk Pharmaceutical Co., Ltd., Beijing, China) and confirmed by a Treponema pallidum particle agglutination (TPPA) assay (Serodia-TPPA, Fujirebio Inc., Tokyo, Japan).

An outreach worker notified the participants to access testing results at the local CDC upon availability (normally within 10 days). Participants with positive HIV or syphilis results received further testing and counseling from the local CDC, and were referred to a local national free anti-retroviral treatment (ART) center, or CDC-affiliated hospitals for syphilis treatment.

### Statistical analysis

Data were checked for accuracy through double data entry in EpiData software (The EpiData Association, Odense, Denmark, version 3.02) and analyzed using SAS software (version 9.1, SAS Institute Inc., Cary, USA).

The prevalence of HIV and syphilis and odds ratios (OR) and 95% confidence intervals (CI) were calculated. Probability values were derived from univariate logistic regression. Variables that had a significant association (P < 0.05) with HIV or syphilis infection in the univariate analysis were included as potential variables in the multivariate logistic regression analysis. Final associated factors were identified provided that they had a significant association (P < 0.05) with HIV or syphilis infection in the multivariate analysis.

### Ethics statement

The study protocol has been reviewed and approved by the Institutional Review Board of the National Center for AIDS/STD Control and Prevention, Chinese Center for Disease Control and Prevention. A written informed consent for participating in the study was obtained from all study participants. A cash stipend of 50 RMB (~8.1 USD) and 30 condoms was given to each participant as compensation for their time.

## Results

### Socio-demographic characteristics

Table 1 describes the characteristics of the study population. There were 1115 low-fee FSWs approached during recruitment who were eligible for the study, and 334(29.9%) refused to participate. Of the 781 study participants, those employed in Guangxi, Hunan, and Yunnan accounted for 39.7% (310), 31.5% (246), 28.8% (225) respectively. The mean age of the participants was 39 years, ranging from 16 to 67 years. Participants aged between 30 and 40, 41 and 50 accounted for 43.5% and 36.5% respectively. Seventy-four percent of participants married or were cohabiting with a male partner. Two-thirds of participants had received less than five years’ education (equivalent to elementary school in China). Most of the participants’ household registrations were non-local (64.7%).

**Table 1:** **Demographic characteristics**, **work related information and HIV**-**related behaviors among low**-**fee female sex workers in 12 cities in China**, **2012**-**2013**

**Variables**	**N**	**%**
**Demographic characteristics**		
**Age (years)**^**a**^		
≤40	460	58.9
>40	321	41.1
**Marital status**		
Married/cohabiting	581	74.4
Single	200	25.6
**Years of schooling**		
>5	230	29.4
≤5	551	70.6
**Ethnicity**		
Han ethnicity	473	60.6
Minorities	308	39.4
**Household registration**		
Non-local	505	64.7
Local	276	35.3
**Province of employment**		
Guangxi	310	39.7
Hunan	246	31.5
Yunnan	225	28.8
**Work-related information**		
**Had ever changed work location**		
Yes	98	12.6
No	681	87.4
**Length of being a FSW**		
<6 months	249	31.9
7-12 months	83	10.6
13-24 months	148	19.0
>24 months	301	38.5
**Soliciting venues**		
Small hair/beauty salons	162	20.7
Small guesthouses	136	17.4
On streets	168	21.5
Self -rented rooms	238	30.5
“Market day” buildings	77	9.9
**Price charged for vaginal sex**		
20-50	633	81.0
≤20	148	19.0
**Estimated average age of clients**		
<50	422	54.0
≥50	359	46.0
**HIV-related behaviors**		
**Consistent condom use with clients in the past 30 days**		
Yes	381	48.8
No	400	51.2
**Injection drug use in the past 6 months**		
Yes	30	3.9
No	751	96.1
**Received HIV testing in the past year**		
Yes	309	39.6
No	472	60.4

^a^The cut-off point of 40 years old was set to distinguish older FSWs from younger FSWs [18,19].

### Work-related information

The majority of participants had never changed work locations since they started working as an FSW. Nearly 40% of participants had worked as an FSW for more than two years. Twenty percent of the participants charged 20 RMB or less for vaginal sex. Half of participants estimated that the age of their clients was 50 years and above.

Participants who solicited clients in their self-rented rooms or in “market day” buildings accounted for 41.4%. Further analysis found that compared with those working in other venues, these participants were significantly more likely to be older (OR: 2.8, 95% CI: 2.1-3.8, P < 0.0001), less educated (OR: 2.2, 95% CI: 1.6-3.0, P < 0.0001), estimated an older age for their clients (OR: 3.5, 95% CI: 21.6-4.7, P < 0.0001), and charged less than 20 RMB for vaginal sex (OR: 3.9, 95% CI :2.7-5.7, P < 0.0001) (data not shown).

### HIV-related behaviors

More than a half of the participants reported no consistent condom use with clients (participants were considered to have used condoms consistently if she used condoms every time with a client in the past 30 days). The three most common reasons for inconsistent condom use were as follows: accepting clients’ refusal to use condoms without charging additional fees due to economic pressure (359/400); clients paying more if participants did not use condoms (52/400); and participants judged that the clients were free of HIV/STIs (53/400).

Four percent of participants reported having ever used injection drugs in the past 6 months, and 43.3% (13/30) of IDUs were based in Kaiyuan city, Yunnan. In the past 12 months, 40% of the participants had received HIV testing.

### Prevalence of HIV and syphilis

Table 2 describes the prevalence of HIV and syphilis and associated factors. All participants were tested for HIV and syphilis. There were 37 HIV positives, accounting for 4.7% of the participating FSWs. HIV prevalence among participants employed in Guangxi, Hunan and Yunnan were 4.2% (13/310), 1.6% (4/246), and 8.9% (20/225) respectively. A total of 117 (15.0%) participants were syphilis infected, with 14.2% (44/310), 11.8% (29/246), and 19.6% (44/225) among participants employed in Guangxi, Hunan, and Yunnan, respectively.

**Table 2:** **Univariate and multivariate analyses of factors associated with HIV and syphilis infection among low**-**fee female sex workers in 12 cities in China**, **2012**-**2013**

**Variables**	**Syphilis infection**						**HIV infection**					
	**N**	**%**	**OR(95% CI)**	**P value**	**AOR(95% CI)**	**P value**	**N**	**%**	**OR(95% CI)**	**P value**	**AOR(95% CI)**	**P value**
**Age (years)**												
≤ 40	44	9.6	1		1		11	2.4	1		1	
>40	73	22.7	2.8(1.9-4.2)	<0.0001	1.8 (1.2-2.9)	0.014	26	8.1	3.6(1.8-7.4)	0.0005	2.6(1.1-6.1)	0.0257
**Marital status**												
Married/cohabiting	77	13.3	1		1		23	7	1			
Single	40	20	1.6(1.1-2.5)	0.022	1.3(0.8-2.1)	0.2399	14	4	1.8(0.9-3.6)	0.0848		
**Years of schooling**												
>5	21	9.1	1		1		9	3.9	1			
≤5	96	17.4	2.1(1.3-3.5)	0.0036	1.5(0.9-2.6)	0.1212	28	5.1	1.3(0.6-2.8)	0.4849		
**Ethnicity**												
Han ethnicity	62	13.1	1				22	4.7	1			
Minorities	55	17.9	1.4(1.0-2.1)	0.07			15	4.9	1.0(0.5-2.1)	0.8881		
**Household registration**												
Non-Local	77	15.2	1				16	3.2	1		1	
Local	40	14.5	0.9(0.6-1.4)	0.7776			21	7.6	2.5(1.3-4.9)	0.0067	3.3(1.5-6.9)	0.0022
**Province of employment**^**a**^												
Guangxi	44	14.2	1		1		13	4.2	1		1	
Hunan	29	11.8	0.8(0.5-1.3)	0.0773	0.7(0.4-1.2)	0.0045	4	1.6	0.4(0.1-1.2)	0.0104	0.3(0.1-0.9)	0.0024
Yunnan	44	19.6	1.5(0.9-2.3)	0.02	2.1(1.2-3.8)	0.0003	20	8.9	2.2(1.1-4.6)	0.0005	2.7(1.1-6.7)	0.0003
**Had ever changed workplace**												
Yes	9	9.2	1				3	3.1	1			
No	108	15.9	1.9(0.9-3.8)	0.0882			34	5	1.7(0.5-5.5)	0.4055		
**Length of being a FSW**												
≤ 2 year	64	13.3	1				17	3.5	1			
>2 year	53	17.6	1.4(0.9-2.1)	0.1042			20	6.6	1.9(1.0-3.8)	0.0505		
**Soliciting venues**^**b**^												
Small hair/beauty salons/guesthouses, streets	48	10.3	1		1		14	3	1		1	
Self- rented rooms/“market day” buildings	76	21.9	2.4(1.6-3.6)	<0.0001	2.3(1.4-3.7)	0.0012	23	7.3	2.5(1.3-5.0)	0.0072	3.9(1.5-10.0)	0.0042
**Price charged for vaginal sex**												
20-50	79	12.5	1		1		22	3.5	1		1	
≤20	38	25.7	2.4(1.6-3.8)	<0.0001	1.2(0.8-1.9)	0.4277	15	10.1	3.1(1.1-6.2)	0.001	2.2(0.9-5.2)	0.0907
**Estimated average age of clients**												
≤50	68	18.9	1		1		18	5	1			
>50	49	11.6	1.8(1.8-2.6)	0.0046	1.2(0.8-1.9)	0.4277	19	4.5	1.1(0.6-2.2)	0.7375		
**Received HIV testing in the past year**												
No	72	15.3	1				16	3.4	1		1	
Yes	45	14.6	0.9(0.6-1.4)	0.7913			21	6.8	2.1(1.1-4.0)	0.0316	1.4(0.7-3.0)	0.3737
**Consistent condom use with clients in the past 30 days**												
Yes	44	11.5	1		1		20	5.2	1			
No	73	18.2	1.7(1.1-2.6)	0.0092	1.6(1.0-2.6)	0.0431	17	4.2	1.2(0.6-2.4)	0.5119		
**Injection drug use in the past 6 months**												
No	110	14.6	1				28	3.7	1		1	
Yes	7	23.3	1.8(0.7-4.2)	0.1992			9	30.9	11.0(4.6-21.3)	<0.0001	13.5(4.5-40.1)	<0.0001
**Syphilis infection**												
No	-	-	-	-			25	3.8	1		1	
Yes	-	-	-	-			12	10.3	2.9(1.4-6.0)	0.0035	1.6(0.7-3.6)	0.2439

^a^Provinces of employment are treated as one of individual characteristic and included as a variable in logistic regression analysis instead of using multi-level modeling. IDUs in Kaiyuan city disproportionately contributed to the HIV prevalence among participants working in Yunnan. After omitting participants of Kaiyuan city from the analysis, HIV prevalence among participants working in Yunnan was 0.6%, the lowest compared with the other two provinces.

^b^The classification of venue type is based on whether or not the participants worked independently. Low-fee FSWs who solicit in self-rented rooms or in “market day” buildings manage their own time and workplace. However, those who solicit in other venues report to a manager.

HIV prevalence was 7.3% among participants who solicit in self-rented rooms and “market day” buildings and 3.0% among participants in all other venues. The prevalence of syphilis was 21.9% among the former and 10.3% among the latter. Among participants who had ever used injection drugs in the past 6 months, HIV prevalence was 30.9%, which was 10 times more than non-injection drug users (non-IDUs). Additionally, all HIV positive IDUs were based in Kaiyuan city.

### Factors associated with HIV infection

Univariate analysis found eight factors associated with HIV infection, namely older age (OR: 3.6, 95% CI: 1.8-7.4), local household registration (OR: 2.5, 95% CI: 1.3-4.9), province of employment (Hunan vs Guangxi OR: 0.4, 95% CI:0.1-1.2; Yunnan vs Guangxi OR: 2.2, 95% CI: 1.1-4.6), soliciting in “self-rented” rooms or “market day” buildings (OR:2.5, 95% CI: 1.3-5.0), lower price charged for vaginal sex (OR:3.1, 95% CI: 1.1-6.2), received HIV testing in the past year (OR:2.1, 95% CI: 1.1-4.0), injection drug use in the past 6 months (OR:11.0, 95% CI: 4.6-21.3) and syphilis infection (OR:2.9, 95% CI: 1.4-6.0).

The final multivariate model identified five factors associated with HIV infection: older age (OR: 2.6, 95% CI: 1.1-6.1), local household registration (OR: 3.3, 95% CI: 1.5-6.9), employed in Yunnan province (OR: 2.7, 95% CI: 1.1-6.7), soliciting in self-rented rooms and “market day” building (OR: 3.9, 95% CI: 1.5-10.0), and injection drug use in the past 6 months (OR: 13.5, 95% CI: 4.5-40.1).

### Factors associated with syphilis infection

Univariate analysis found eight factors were associated with syphilis infection: older age (OR: 2.8, 95% CI: 1.9-4.2), single (OR: 1.6, 95% CI: 1.1-2.5), less years of schooling (OR: 2.1, 95% CI: 1.3-3.5), employed in Hunan or Yunnan provinces (Hunan vs Guangxi OR: 0.8, 95% CI: 0.5-1.3, NS; Yunnan vs Guangxi OR:1.5, 95% CI: 0.9-2.3), soliciting in self-rented rooms and “market day” building (OR: 2.4, 95% CI: 1.6-3.6), lower price charged for vaginal sex (OR: 2.4, 95% CI: 1.6-3.8), higher estimated average age of clients (OR: 1.8, 95% CI: 1.8-2.6) and no consistent condom use with clients in the past 30 days (OR: 1.7, 95% CI: 1.1-2.6).

The final multivariate model identified four factors associated with syphilis infection: older age (OR: 1.8, 95% CI: 1.2-2.9), employed in Yunnan province (OR: 2.1, 95% CI: 1.2-3.6), soliciting in self-rented rooms and “market day” buildings (OR: 2.3, 95% CI: 1.4-3.7), and no consistent condom use with clients in the past 30 days (OR: 1.6, 95% CI: 1.0-2.6).

## Discussion

The overall prevalence of HIV and syphilis among low-fee female sex workers in our study were 4.7% and 15.0%, respectively. Compared with previous studies reporting HIV and syphilis prevalence among FSWs, the prevalence found in our study were about twice higher [6,15]. There is an alarmingly high prevalence of HIV and STI that still exist among low-fee FSWs despite extensive HIV prevention efforts targeting FSWs over the past decades.

The prevalence of HIV was distributed differently among participants working in different provinces. Compared with Guangxi and Hunan, participants employed in Yunnan exhibited the highest prevalence (8.9%). However, IDUs in Kaiyuan city disproportionately contributed to the prevalence. Of the 30 IDUs in our study, 13 IDUs (43%) and all of the HIV positive IDUs were based in Kaiyuan city. Previous studies also found high levels of HIV infection among FSWs in this city [14,20-22]. After omitting participants of Kaiyuan city from the analysis, HIV prevalence among participants working in Yunnan was 0.6%, the lowest compared with the other two provinces. Hence, intervention strategies among low-fee FSWs working in Yunnan should focus on cities with sizeable populations of low-fee FSWs exchanging sex for drugs, and enroll them into methadone maintenance treatment and needle exchange programs [23].

We found that among low-fee FSWs, differences in HIV and syphilis prevalence were attributable to venue type. Participants who solicit in self-rented rooms and “market day” buildings were twice as likely to test positive for both HIV and syphilis infection than those soliciting in other venues. Additionally, the former were more likely to be older, less educated, estimated an older age for their clients and charged less than 20 RMB for vaginal sex. They may lack self-perceived risk and knowledge of HIV/STI, and may be unable to charge more for vaginal sex due to decreased desirability resulting from older age [24,25]. These FSWs may therefore be under more pressure to not use condoms when requested by a client, despite the high risk of HIV/STI infection. In addition, these FSWs work independently, are not controlled nor protected by gatekeepers, and isolated from their peers, which renders the influence of gatekeepers and peers to promote consistent condom use unfeasible [26,27]. Intervention design and implementation must take into account these differences between subgroups of low-fee FSWs and prioritize specifically targeting low-fee FSWs who solicit in self-rented rooms and “market day” buildings.

The reported rate of consistent condom use in the past 30 days was 50% in our study, which was lower than in other FSW studies in China [5,28]. The actual rate of condom use may be even lower since participants may have self-reported an inflated condom use rate [29]. Inconsistent condom use increases the infection risk of STI like syphilis, which facilitates HIV transmission [30,31]. Accordingly, we found that low-fee FSWs who did not use condoms consistently were nearly twice likely to be syphilis infected. When we explored why condom use did not always occur, the most common reason was that in order to earn enough money, FSWs often accepted clients’ refusal to use condoms. Like other groups of low-wage migrant women, many low-fee FSWs face the economic pressure of supporting a family [13,32], which may be a factor determining condom use with clients [3,33,34]. Client refusal of condom use may be attributed to two possible explanations. First, nearly half of the clients were estimated by study participants to be 50 years old and above, and it is likely that many clients experienced difficulty using condoms due to erectile dysfunction [35]. Second, previous studies have found that clients of low-fee FSWs have insufficient knowledge and risk perception of HIV/STI infection and may be unlikely to view condom use as necessary [36,37].

Consistent condom use should be promoted through interventions that take into account the specific context of risk behavior for low-fee FSWs. Efforts to increase awareness of consistent condom use should be coupled with tactics that do not negatively impact FSWs’ earnings and environmental-level supports [38]. Successful negotiations of condom use such as helping the client to maintain an erection, or persuading the clients through disease fear arousal [39,40], can be considered among low-fee FSWs, especially those soliciting in self-rented rooms and “market day” venues. Moreover, for low-fee FSWs working in venues, environmental supports like requiring managers of venues to make condoms available on-site and funding free condoms to FSWs may also decrease risk behavior [34,38,41].

HIV-positive low-fee FSWs often continue to be actively involved in commercial sex [42]. Early detection and treatment is needed to decrease risk of HIV transmission. In our study, we found that although two thirds of low-fee FSWs had a non-local household registration, they did not change their work location frequently. Moreover, HIV infection is three times more likely to be detected among low-fee FSWs with local household registration compared to non-local FSWs. These findings indicate that low-fee FSWs migrate infrequently and that venue-based testing and further ART management may be feasible. To promote HIV testing, confidential and accessible testing technologies can be implemented in venues directly, such as rapid testing and mobile testing vans, which have successfully reached other hidden, vulnerable populations [43-45]. These measures need to be coordinated with other entities, including the local government and public security bureaus. Despite policies maintaining confidentiality, in practice, sex workers are often compelled to leave the venue or detained in detention centers upon detection of HIV, and these practices may cause FSWs to refuse HIV testing or migrate elsewhere [46]. Local governments should develop and implement supportive policies to work with HIV positive sex workers and prioritize linking them to national free ART [14].

There are several limitations in our study. First, the overall sample size in our study is somewhat small compared to other FSW studies. However, the sample is still reasonably representative since it targets only low-fee FSWs, and experienced local outreach workers are familiar with the community in each study site mapped and approached all low-fee venues, including self-rented rooms and “market day” building which have rarely been described in the public health literature on Chinese FSWs before. These results highlight the need for further research on implementing interventions targeting specific low-fee FSW groups. Second, HIV and syphilis prevalence among low-fee FSWs who solicit in self-rented rooms or “market day” venues may be underestimated. The primary reason was that these venues were sometimes not recognizable to outsiders, whereas venues like guesthouses and hair/beauty salons were more easily accessible since they have obvious markers of commercial sex. Thirdly, information biases particularly those related to condom use may exist in our study, despite the extensive training on survey administration and working with sex workers that study staff received. The rate of condom use may be inflated due to desire for conformity or stigma. To confirm self-reported condom use, questions on condom availability and correct condom usage can be added in future studies.

## Conclusions

Our study provided further evidence that of a higher prevalence of HIV and syphilis among low-fee FSWs. Substantial heterogeneity also exists within this population. Low-fee FSWs who work in self-rented rooms or solicit during “market day” had the highest prevalence of HIV and syphilis. Further studies should assess the demographics of this group and the epidemiologic characteristics of HIV and other STI transmission to inform intervention strategies targeting this subpopulation. Moreover, although the total number of IDUs was small, they were concentrated in Kaiyuan city in Yunnan and had a much higher HIV prevalence compared with non-IDUs. Therefore, interventions for IDUs like MMT and needle exchange should be also integrated in cities where low-fee FSWs are at dual risk of HIV infection. Given the high risk of HIV and syphilis for low-fee FSWs across all twelve of our study sites, multifaceted approaches to HIV prevention for this group, especially those that link FSWs to testing and treatment, are critical to controlling infections in areas with expanding epidemics among FSWs.

## Competing interests

The authors declare that they have no competing interests.

## Authors’ contributions

KR and ZW designed the study. CZ, YW, WD, YZ, XC, MJ, WL, JZ, MY, LY helped coordinate the implementation of the field survey. CZ did primary data analysis. CZ and WMD interpreted the data and drafted the paper. All authors contributed to the interpretation of the data, critically reviewed several versions of the manuscript, and approved the submitted version of the report.

## Authors’ information

***China National HIV Prevention Study Group*** Zunyou Wu (NCAIDS, China CDC), Keming Rou (NCAIDS, China CDC), Na He (Fudan University), Jie Xu (NCAIDS, China CDC), Yurong Mao(NCAIDS, China CDC), Jianhua Li (Yunnan Institute of Drug Abuse), Xiangdong Min (Yunnan CDC), Fan Li (Xinjiang CDC), Wei Liu (Guangxi CDC), Linlin Zhang (Sichuan CDC), Limei Shen (Guizhou CDC), Minghua Zhuang (Shanghai CDC), Liangui Feng (Chongqing CDC), Hongbo Zhang (Anhui Medical University), Yehuan Sun (Anhui Medical University), and Weihua Cao (Peking University) served as the Research Steering Committee. Keming Rou (NCAIDS, China CDC), Chu Zhou (NCAIDS, China CDC), Wei Dong (NCAIDS, China CDC), Yu Wang (NCAIDS, China CDC), Yuejiao Zhou (Guangxi CDC), Xi Chen (Hunan CDC), Manhong Jia (Yunan CDC), Wei Liu (Guangxi CDC), Jun Zheng (Hunan CDC), Yangling Ma (Yunnan CDC), and Youfang Li (Yunnan CDC) served as the Research Implementation Team for intervention targeting female sex workers. Hongbo Zhang (Anhui Medical University), Yehuan Sun (Anhui Medical University), and Weihua Cao (Peking University) served as the Quality Control and Quality Assurance Team.

Chu Zhou and Keming Rou contributed equally as joint first authors.

## Pre-publication history

The pre-publication history for this paper can be accessed here:

http://www.biomedcentral.com/1471-2334/14/225/prepub
